# Evaluation of Thiol/Disulfide Homeostasis in Bronchiectasis

**DOI:** 10.1155/2022/8340450

**Published:** 2022-01-29

**Authors:** Songul Ozyurt, Neslihan Ozcelik, Bilge Yilmaz Kara, Medeni Arpa, Yavuz Metin, Ozcan Erel, Salim Neselioglu

**Affiliations:** ^1^Recep Tayyip Erdogan University, Training and Research Hospital, Chest Disease, Rize, Turkey; ^2^Recep Tayyip Erdogan University, Training and Research Hospital, Medical Biochemistry, Rize, Turkey; ^3^Ankara University, Medical Faculty Ibn i Sina Hospital, Department of Radiology, Ankara, Turkey; ^4^Yildirim Beyazit University, Faculty of Medicine, Department of Medical Biochemistry, Ankara, Turkey

## Abstract

**Purpose:**

Thiols are sulfhydryl-containing organic compounds that have an important role in preventing cellular oxidative stress. This study compares the blood oxidative stress marker levels in bronchiectasis cases during their stable periods with healthy controls.

**Materials and Methods:**

Seventy-seven patients (49 patients with stable bronchiectasis/28 healthy controls), followed up by the chest disease clinic, were included in the study. Peripheral blood thiol-disulfide parameters (NT: native thiol (−SH); TT: total thiol (−SH + SS); SS: disulfide (−SS); SS-SH: disulfide/native thiol index; SS-TT: disulphide/total thiol index; SH-TT: native thiol/total thiol index), and ischemia-modified albumin (IMA) levels were examined in the stable bronchiectasis group and the control group. Thiol-disulfide homeostasis was evaluated using a novel and automated assay. *Findings and Result*. Blood native thiol levels in patients with stable bronchiectasis were found to be significantly higher compared with healthy controls. A positive correlation between the total airway disease score and IMA levels was present. Our findings revealed that native thiol levels, which constitute a part of the antioxidant defense system, are increased in patients with stable bronchiectasis.

## 1. Introduction

Bronchiectasis is a chronic airway disease characterized by abnormal degradation and widening of large airways, that is, bronchi and bronchioles. Etiologies of bronchiectasis include cystic fibrosis, primary ciliary dyskinesia, genetic anomalies, immune deficiency syndromes, modified host defense and autoimmune diseases, significant infections, and acquired disorders [1]. In bronchiectasis, airways show gradual damage due to proteinase enzymes secreted by epithelial cells, neutrophil-produced reactive oxygen radicals, eosinophils, macrophages, chronic microbial infection, airway inflammation, and interaction of substances harming the tissues [1].

It is produced due to the normal metabolism or various reasons such as ischemia-reperfusion, aging, radiation, high oxygen pressure, inflammation, and exposure to chemical agents, and oxygen free radicals [2] cause metabolic, structural, and functional damages, which may lead to cell death by reacting to various cellular components and macromolecules. An excessive number of free radicals produced during inflammation repress the antioxidant defense systems, which leads to considerable damage in the airway epithelial cells and other airway structures [3]. Oxidative stress occurs when the production of harmful oxidants exceeds the detoxification capacity of the antioxidant defense system in the body.

Various studies suggest that oxidative stress potentially contributes to the pathogenesis of many respiratory diseases, including cystic fibrosis, pulmonary tuberculosis, and acute respiratory distress syndrome [4]. Furthermore, it has been shown that pulmonary diseases with chronic airway inflammation, such as chronic obstructive pulmonary disease (COPD) and bronchial asthma, increase oxidative stress indicators in the plasma [5, 6].

Thiol is an organic compound containing a sulfhydryl group that plays a critical role in preventing cellular oxidative stress. Cysteine, through its functional thiol group, plays an important role in the prevention of oxidative damage in the body [7].

### 1.1. Biological Significance of Thiols and Disulfides

Thiols and disulfides play an important role in the stabilization of protein structures and regulation of protein and enzyme functions, receptors, carriers, Na-K channel, and transcription. The dynamic thiol-disulfide homeostasis (TDH) is critical for antioxidant defense, detoxification, apoptosis, regulation of enzyme activities, transcription, and cellular signal transduction mechanisms [7]. Abnormal TDH contributes to the pathogenesis of various diseases, such as diabetes mellitus, cardiovascular diseases, malignancy, rheumatoid arthritis, chronic renal failure, Parkinson's disease, Alzheimer's disease, multiple sclerosis, and liver diseases [7]. Measuring the TDH involves assessing the native thiol [-SH](NT), dynamic disulfide [-S-S-] (DS), and total thiol [(-SH)+(-S-S-)](TT) levels and the dynamic “-SH/-S-S-” homeostasis [7].

### 1.2. Ischemia-Modified Albumin (IMA)

Ischemia-modified albumin (IMA) is an albumin form that displays ischemic and oxidative damage and goes through structural changes. It is an indicator that might be used in many cases, such as strokes and cerebrovascular accidents, mesenteric ischemia, myocardial ischemia, and musculoskeletal ischemia [8].

This study provides an assessment of the thiol-disulfide homeostasis, an indicator of the activity of the antioxidant system, and IMA levels, indicating oxidative damage, in patients with stable bronchiectasis without acute inflammation symptoms.

## 2. Materials and Methodology

Forty-nine patients diagnosed with stable bronchiectasis without acute infection symptoms or findings were included in the study. The control group included 28 healthy individuals of the same age and sex. The age, sex, and high-resolution computed tomography (HRCT) findings of all patients were recorded. Peripheral blood samples (total, 4 mL) were collected from the antecubital vein in heparinized tubes and were stored at 2°C–4°C for measuring the thiol-disulfide hemostasis parameters. The blood was centrifuged at 1500 g for 10 min to separate the plasma. The separated plasma was then stored at −80°C until analysis. The ethical committee of the Department of Medicine of Recep Tayyip Erdogan University approved the study (decision no. 2021/92).

### 2.1. Inclusion Criteria for the Study

The inclusion criteria are as follows:

- Being older than 18 years
- Detection of bronchiectasis on the HRCT scan
- Being in a stable condition during the course of the disease (stability was defined as the absence of acute inflammation and antibiotic use within four weeks before the application; absence of acute inflammation was defined as the lack of the following symptoms: absence of an increase in dyspnea, cough and sputum volume, or purulence)
- Having no smoking habits
- Not having chronic diseases such as hypertension, diabetes mellitus, chronic kidney disease, and coronary artery disease, malignancy, or rheumatological diseases

### 2.2. Exclusion Criteria

The exclusion criteria are as follows:

- Patients under 18 years of age
- Patients with acute exacerbation of bronchiectasis (an increase in dyspnea, cough and sputum volume, or purulence)
- Patients with an infectious or inflammatory condition (infections in other parts of the body, e.g., urinary tract infection and inflammatory arthritis)

### 2.3. Diagnosing and Scoring Bronchiectasis

Bronchiectasis was diagnosed using HRCT. Normally, airways are not visible in the lung parenchyma within 1 cm of the costal pleura. Furthermore, the inner diameter of the bronchi is equal to that of the accompanying pulmonary artery within the same bifurcation level. In adults, the ratio of the bronchoarterial diameter being lower than one is considered abnormal, and the visibility of the peripheral airways within 1 cm in the costal pleura is considered in favor of a bronchiectasis diagnosis [1]. The presence and prevalence of bronchiectasis were examined in accordance with the findings from the HRCT scans of the patients, and each lobe was evaluated individually based on the Bhalla scoring system [9]. Based on this classification, the bronchiectasis rate was scored as follows: 0: none; 1: less than 25% of the lobe volume; 2: between 25% and 50% of the lobe volume; and 3: more than 50% of the lobe volume. The total score for each patient was calculated as the bronchiectasis severity score. Furthermore, based on the findings from the HRCT scans, wall thickness was evaluated with the following scores: 0: no thickening; 1: thickening between 25% and 50%; 2: thickening of more than 50%; and 3: completely obliterated. Furthermore, accompanying symptoms, such as peribronchial thickening, small airway pathologies, and mosaic perfusion in the neighboring lung, were also evaluated and recorded using HRCT.

### 2.4. Thiol-Disulfide Homeostasis Parameters

Thiol-disulfide homeostasis parameters were measured using a novel automatic and spectrophotometric method described by Erel and Neselioglu [10]. Free functional thiol groups (-SH) were extricated by decreasing disulfide bonds (-S-S-) using sodium borohydride (NaBH4). Unused NaBH4 remnants were completely removed using formaldehyde. This prevented further reduction of 5,5′-dithiobis-2-nitrobenzoic acid (DTNB) as well as any disulfide bonds resulting from possible reactions with DTNB. Total thiol groups including reduced and native thiol groups were determined after reactions with DTNB. The disulfide parameter value can be calculated as half of the native thiol content and total thiol content. Disulfide/total thiol, disulfide/native thiol, and native thiol/total thiol ratios were calculated.

IMA levels were analyzed using the rapid and colorimetric method defined by Bar-Or et al. [11].

### 2.5. Statistical Analysis

SPSS 17.0 (Chicago Inc., 2008) and MedCalc® Statistical Software version 19.8 (MedCalc Software Ltd, Ostend, Belgium; https://www.medcalc.org; 2021) were used for the statistical analysis. Categorical variables were indicated as frequency (*n*) and percentage (%). The fitness of the continuous variables to normal distribution was tested using the Kolmogorov–Smirnov method. The NT and TT levels, identified to display a normal distribution, were indicated as mean and standard deviation, while other laboratory parameters (disulfide, SS-SH, SS-TT, SH-TT, and IMA) were indicated as median and interquartile range as they do not display a normal distribution. Student's *t*-test and Mann–Whitney *U* tests were used for the binary comparison of the continuous variables. As for the categorical variables, they were analyzed using the Pearson chi-squared and Fisher's exact tests. The correlation between bronchiectasis and oxidative stress parameters was analyzed using Spearman's correlation test. *p* < 0.05 was accepted for significance.

## 3. Findings

The data for 77 participants (49 patients with stable bronchiectasis and 28 healthy controls) were compared. The mean age was 58.8 years; 51.9% of the participants (*n* = 40) were females, and 48.1% (*n* = 37) were males. Both groups were found to be similar in terms of age and sex distribution (*p* > 0.05; Table 1).

**Figure 1:**
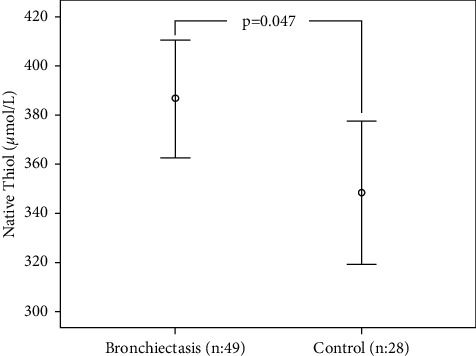
Comparison between the native thiol levels in patients with bronchiectasis and the control group with an error bar graph.

The mean NT levels in the blood were found to be significantly higher in patients with stable bronchiectasis compared with healthy controls (*p*=0.047; Figure 1). Other TDH parameters (mean total thiol levels, median level distributions of disulfide, SS-SH, SS-TT, and SH-TT indices) and IMA were found to be similar for both groups (*p* > 0.05; Table 1).

The examination of the prevalence of bronchiectasis revealed that the disease occurred bilaterally in multiple lobes in 53.1% of the patients, making it the most frequent incidence.  Table 2 shows the other variables concerning the disease.

**Figure 2:**
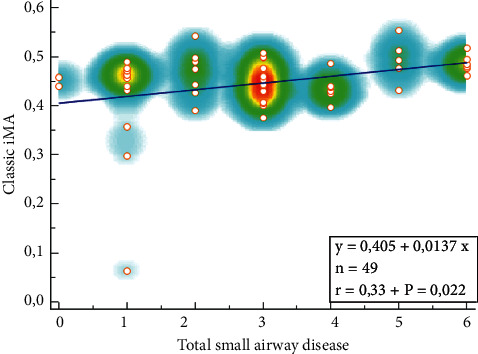
The correlation between the IMA levels in the blood and total small airway disease scores.

The correlation between the thiol-disulfide indices and IMA levels and bronchiectasis disease scores was also examined. A positive, medium-level, and statistically significant correlation was identified between the IMA levels in the blood and the total small airway disease scores (rho = 0.300; *p*=0.036). No significant correlation was found between the other parameters and the variables concerning bronchiectasis (*p* > 0.05 for all) (Table 3). The correlation between the IMA levels in the blood and total small airway disease scores is shown in Figure 2 (*r* = 0.33, *p*=0.022).

## 4. Discussion

Our study reported higher NT levels in patients with stable bronchiectasis compared with healthy controls. Furthermore, a positive correlation between the total airway disease scores calculated for bronchiectasis and IMA levels was detected.

Reactive oxygen species (ROS), such as the hydroxyl radical, superoxide radical, and hydrogen peroxide, are produced during cellular metabolism. The insufficiency of antioxidants protecting the organism from the adverse effects of these substances disrupts the oxidative balance, causing oxidative stress. Elevated ROS levels in oxidative stress causes cell damage and cellular death by harming macromolecules within cells, such as lipids, proteins, and DNA [12]. As is the case in many diseases, oxidative stress plays an important role in the pathogenesis of various pulmonary diseases, such as COPD, asthma, pneumonia, tuberculosis, and bronchiectasis [13]. Previous studies reported significant increases in the oxidants in patients with lung diseases characterized by chronic inflammation such as bronchiectasis [5, 14, 15].

As for thiols, they constitute an important component of the plasma antioxidant system, where they reverse the effects of the oxidants and protect the organism from their harmful effects [7]. The literature review did not reveal any studies assessing TDH in bronchiectasis. Although existing studies in the literature seem to concentrate on the increase in the oxidant capacity, there are also studies reporting an increase in antioxidant capacity against increased oxidation [16]. In their study conducted on pediatric patients with bronchiectasis, Gedik et al. demonstrated that chronic inflammation also increases the total antioxidant capacity (TAC) and total oxidant status (TOS) [3]. In a study conducted on patients with asthma, Cakmak et al. reported increases in both TAC and TOS levels [17]. Being in line with the results of these studies, we also reported higher native thiol levels in our patients with stable bronchiectasis compared with the healthy control group, which shows that the antioxidant system may be functional in patients with stable bronchiectasis.

Metals such as cobalt, copper, and nickel are connected to the last amino-terminal region in the albumin structure. In cases such as hypoxia, acidosis, and free-radical damage, the connection of these metals is diminished, which changes the albumin structure, producing “ischemia-modified albumin” (IMA) [18]. It was reported that, in many chronic inflammatory pulmonary diseases and hypoxic cases (pleural effusion, pulmonary embolism, etc.), serum IMA levels increase [19–24]. Furthermore, IMA was also reported to reflect disease severity. Our study examines IMA levels as one of the indicators of oxidative stress. When comparing the results with those of healthy controls, they seem to be similar. However, a positive correlation was spotted upon examining the relationship with the small airway disease score. Inflammation is expected to be more intense during the acute inflammation period. The lack of differences between the IMA levels in our patients and healthy controls may be attributed to the stability of our patients. Alternatively, the positive correlation with the calculated total small airway disease score indicates a potential relationship with disease severity.

### 4.1. Limitations of the Study and Suggestions for Future Studies

The basic limitations of our study include its monocentral nature and the small number of patients. The study was conducted on patients with stable bronchiectasis. Since patients in the acute exacerbation period were excluded, the study excludes any results regarding the state of the antioxidant system in these patients. Further studies including both types of patients are required.

## 5. Conclusions

Our study demonstrated that, in bronchiectasis, thiol levels were increased, which indicated that the antioxidant system is active. It should be noted that, in bronchiectasis, substantial evidence is emerging suggesting persistent chronic inflammation in the stable period as well.

Considering our study and previous studies, the need for approaches supporting the antioxidant system in addition to treatments targeting infections becomes evident.

## Figures and Tables

**Table 1:** Comparison between the data of patients with stable bronchiectasis (*n* = 49) and the healthy control group (*n* = 28).

	Total	Bronchiectasis	Control	Statistic	*p* value
	*n* = 77	*n* = 49	*n* = 28	T, *z*, or *χ*^2^	
Age (years)^a^	58.8 ± 13.9	57.7 ± 13.1	60.6 ± 15.4	−0.864	0.390
Sex, *n* (%)				0.067	0.796
Female	40 (51.9)	26 (53.1)	14 (50.0)		
Male	37 (48.1)	23 (46.9)	14 (50.0)		
Laboratory					
Native thiol (*μ*mol/L)^a^	372.9 ± 81.9	386.8 ± 83.0	348.4 ± 75.3	2.017	0.047
Total thiol (*μ*mol/L)^a^	405.1 ± 84.4	418.6 ± 85.9	381.3 ± 77.4	1.900	0.061
Disulfide (*μ*mol/L)^b^	15.20 ± 8.15	14.50 ± 9.07	15.92 ± 7.04	−0.699	0.485
SS-SH index (%)^b^	4.013 ± 2.18	3.788 ± 1.96	4.489 ± 2.05	−1.641	0.101
SS-TT index (%)^b^	3.714 ± 1.85	3.521 ± 1.68	4.119 ± 1.72	−1.641	0.101
SH-TT index (%)^b^	92.57 ± 3.70	92.95 ± 3.36	91.76 ± 3.44	−1.641	0.101
Classic IMA (AU)^b^	0.457 ± 0.06	0.457 ± 0.05	0.460 ± 0.07	−0.752	0.452

^a^Arithmetic mean (standard deviation). ^b^Median (interquartile range). ^*∗*^Fisher's exact test. NT: native thiol (-SH); TT: total thiol (-SH + SS); SS: disulfide (-SS); IMA: ischemia-modified albumin; AU: absorbance unit; SS-SH: disulfide/native thiol index; SS-TT: disulfide/total thiol index; SH-TT: native thiol/total thiol index.

**Table 2:** Disease-related features of patients with stable bronchiectasis (*n* = 49).

	Results
Bronchiectasis, *n* (%)	
Right, single lobe	5 (10.2)
Left, single lobe	3 (6.1)
Bilateral, in one lobe each	7 (14.3)
Right, in multiple lobes	4 (8.2)
Left, in multiple lobes	4 (8.2)
Bilateral, in multiple lobes	26 (53.0)
Total	49 (100.0)
Total BE score^b^	5 ± 4.5
Total wall thickness (mm)^a^	5.8 ± 3.7
Total small airway disease score^b^	3 ± 2.5
Total mosaic perfusion^b^	2 ± 3

^a^Arithmetic mean (standard deviation). ^b^Median (interquartile range).

**Table 3:** Analyzing the correlation between the oxidative stress parameters in the bronchiectasis group (*n* = 49) with disease scores.

		Total bronchiectasis score	Total wall thickness	Total small airway disease score	Total mosaic perfusion score
Native thiol (-SH)	Rho	0.01	0.01	−0.08	−0.03
	*p* value	0.96	0.93	0.59	0.83
Total thiol (-SH + SS)	Rho	0.03	0.01	−0.05	−0.01
	*p* value	0.83	0.96	0.72	0.95
Disulfide (-SS)	Rho	0.12	0.09	0.12	0.15
	*p* value	0.34	0.53	0.43	0.30
SS-SH index	Rho	0.17	0.13	0.16	0.18
	*p* value	0.24	0.39	0.26	0.21
SS-TT index	Rho	0.17	0.13	0.16	0.18
	*p* value	0.24	0.39	0.26	0.21
SH-TT index	Rho	−0.17	−0.13	−0.16	−0.18
	*p* value	0.24	0.39	0.26	0.21
Classic IMA	Rho	0.01	−0.08	**0.30** ^*∗*^	.25
	*p* value	0.95	0.58	**0.04**	.09

IMA: ischemia-modified albumin; SS-SH: disulfide/native thiol index; SS-TT: disulfide/total thiol index; SH-TT: native thiol/total thiol index. ^*∗*^: correlation is significant at the 0.05 level (2-sided).

## Data Availability

The data that support the findings of this study are available from the corresponding author upon reasonable request.
